# Supplementary material for: Quantifying heterogeneities in arbovirus transmission: Description of the rationale and methodology for a prospective longitudinal study of dengue and Zika virus transmission in Iquitos, Peru (2014–2019)
Source: PLoS One. 2023 Feb 2;18(2):e0273798. doi: 10.1371/journal.pone.0273798 (PMC9894416; doi:10.1371/journal.pone.0273798)
Supplement: S1 Appendix — This appendix has been provided by the authors to give readers additional information about their work. (DOCX) [file pone.0273798.s001.docx]

Supplementary Appendix

This appendix has been provided by the authors to give readers additional information about their work.

Supplement to: Morrison AC, Paz Soldan VA, Vazquez-Prokopec, GM, et al. **Quantifying Heterogeneities in Arbovirus Transmission: A Prospective Longitudinal Study of Dengue and Zika Viruses in Iquitos, Peru**

Contents

[1 Study Design 5](#_Toc68638146)

[1.1 Community-based cohort 5](#_Toc68638147)

[1.1.1 Acute dengue and Zika virus infections. 5](#_Toc68638148)

[1.2 Longitudinal Cohort 5](#_Toc68638149)

[1.3 Clinic-based febrile surveillance 6](#_Toc68638150)

[1.4 Contact cluster investigations 6](#_Toc68638151)

[2. Blood and other human sampling procedures 6](#_Toc68638152)

[2.1 Collection of Biological Samples 6](#_Toc68638153)

[2.1.1 Venipuncture 6](#_Toc68638154)

[2.1.2 Capillary Samples 7](#_Toc68638155)

[2.2 Other body fluids sampled for suspected Zika cases. 7](#_Toc68638156)

[2.2.1 Urine Samples 7](#_Toc68638157)

[2.2.2 Saliva sample collection 7](#_Toc68638158)

[2.2.3 Breast Milk and Semen. 7](#_Toc68638159)

[2.3 Sample Transport and storage 7](#_Toc68638160)

[3. Entomological surveys 7](#_Toc68638161)

[3.1 Larval and pupal collections 8](#_Toc68638162)

[3.2 Adult captures 8](#_Toc68638163)

[3.3 Establishment of the Genetically Diverse Laboratory *Aedes aegypti* strain 8](#_Toc68638164)

[4. Laboratory Assays 9](#_Toc68638165)

[4.1 Taqman Real-Time PCR for DENV serotypes 1-4. 9](#_Toc68638166)

[4.2 Nested reverse transcription polymerase chain reaction (RT-PCR) for dengue serotypes 1–4. 10](#_Toc68638167)

[4.3 Quantitative Real-Time PCR for ZIKV. 10](#_Toc68638168)

[4.4 IgM capture ELISA 10](#_Toc68638169)

[4.5 Serotype-specific microneutralization enzyme immunoassay (MNT). 10](#_Toc68638170)

[5. Direct Blood Feeding Experiments on DENV/ZIKV-positive participants 11](#_Toc68638171)

[5.1 Blood feeding Procedures. 11](#_Toc68638172)

[5.2 Mosquito processing workflow 11](#_Toc68638173)

[6. Project 2 Interviews and Procedures 11](#_Toc68638174)

[6.1 Full Retrospective Movement Survey 11](#_Toc68638175)

[6.2 24-hour RMS (“24hr RMS”) 12](#_Toc68638176)

[6.3 Dengue Illness Perceptions and Responses (IPR) survey 12](#_Toc68638177)

[6.4 Health Related Quality of Life (HRQoL) survey 12](#_Toc68638178)

[6.5 Costs survey 12](#_Toc68638179)

[6.6 GPS monitoring 12](#_Toc68638180)

[6.7 Temperature Monitoring 12](#_Toc68638181)

[7. References 12](#_Toc68638182)

# 1 Study Design

## 1.1 Community-based cohort

We established a febrile illness surveillance cohort involving ~7,000 participants. We aimed to sustain this number during January 2015 by recruiting participants from 2 ongoing cohort studies in well characterized neighborhoods (Maynas and Tupac Amaru).^1–7^ Concurrently, we recruited DENV/ZIKV cases from a larger randomized vector control intervention trial consisting of 26 clusters of households (~140 houses each).^8^ Between both approaches, ~20,000 Iquitos residents were under disease surveillance.

Enrollment was accomplished by door-to-door visits by nurse technicians, who provided a verbal explanation of the project and left written information sheets with the adult household members. Later, staff carried out a written census that included the name, birthdate, gender, length of residence in the household, and occupation of all household members willing to participate in the study. After inclusion in the study database, field teams attempted to visit all participating households 2 to 3 times per week to detect acute illness (see section below).

A mobile Android application was used to record the outcomes of wellness visits, including logging contact with a household resident (successful visit) or no contact (unsuccessful visit when nobody was home) and periodically ask about the status of each resident registered in the household. This information was used to track active surveillance and days lost-to-follow up (LTFU) for each participant. Only active surveillance days were summed to calculate person-time at risk of infection.

### 1.1.1 Acute dengue and Zika virus infections.

Febrile participants, defined as persons with an oral temperature of ≥ 38^o^C or an axillary temperature of ≥ 37.5^o^C for < five days of duration at the time of visit, were identified through door to door visits to cohort member houses carried out 2-3 x per week including follow up visits the same day to houses where residents were not home and phone calls to participants that requested that approach Study inclusion criteria were modified in May 2016 to capture people with suspected ZIKV infections; i.e., absence of fever but presence of rash, arthralgia, arthritis, or non-purulent conjunctivitis. Participants meeting these criteria provided acute and convalescent (14–21-d interval) serum samples and were monitored clinically each day; i.e., interview, vital sign check, and tourniquet testing. For suspected ZIKV cases who consented, whole blood, urine, semen, breast milk, conjunctival, and/or saliva samples were solicited. Acute serum samples were tested by TaqMan PCR and/or NS1 (SDbioline dengue duo) for DENV and ZIKV. Acute- and convalescent-phase blood samples were screened for anti-DENV IgM antibody by IgM-capture enzyme-linked immunosorbant assay (ELISA).

1.2 Longitudinal Cohort

From the community-based population, all children 2-18 years of age were invited to provide annual blood samples. Sera were tested by a microneutralization assay (MNT) for neutralizing antibodies against all four DENV serotypes and, subsequently, ZIKV. Although we attempted to retain longitudinal participants for the course of the study, new participants were enrolled each year to replace individuals lost to follow up. Our target was to always maintain a longitudinal cohort of 1,500 participants during the study.

## 1.3 Clinic-based febrile surveillance

Iquitos residents aged > 5 years who presented at any of two Ministry of Health hospitals or seven health centers with acute, undifferentiated fever (≥ 38°C or reported use of antipyretics with no clear respiratory, gastrointestinal or urogenital focus) of < 5 days were invited to participate^9^. A study nurse introduced mosquito feeding experiments and disease perception and behavioral studies at the time of enrollment and asked if the enrollee was willing to participate in an additional study if found positive for DENV or ZIKV infection.

## 1.4 Contact cluster investigations

Index cases for contact cluster investigations were identified through the community- and clinic-based surveillance methods described above. After an index case was confirmed positive for DENV or ZIKV, a retrospective movement survey^1^ was administered. The survey was developed^1^ to identify routinely visited residential sites that the index case had visited in the 15 days prior to onset of symptoms. At these locations, all residents > 5 years of age were invited to participate in a cluster investigation. For ZIKV, permission to initiate cluster investigations was based on clinical and epidemiological suspicion of infection. Blood was drawn from each participant at enrollment. Cluster participant’s houses were then visited daily to identify additional participants with DENV or ZIKV infection. An additional blood sample was requested from additional participants. When a person with a DENV or ZIKV infection was identified, blood samples were requested from all participants in the household as soon as possible, but > 2 days after the previous sample. If another participant tested positive, household residents would be resampled until a maximum of 5 blood samples per person was obtained within a 30-day follow up period. In the case where no contacts tested positive after the first sample in a cluster, a second set blood samples were drawn from each participant 30 days after the first blood sample.

# 2. Blood and other human sampling procedures

## 2.1 Collection of Biological Samples

### 2.1.1 Venipuncture

All blood samples were collected in Vacutainer® collection tubes at the home of the participant or in a clinic facility, labeled, and stored in small portable ice chests until they were transported to our field laboratory, i.e., within 4 hours. For the DENV/ZIKV viremic participants, blood and mosquitoes were transported immediately to the NAMRU-6 field laboratory for processing. Samples without additive were centrifuged for 10 minutes at 3,000 rpm at 4^0^C, and sera were then transferred to cryovials and stored at -80^0^C until PCR testing in the NAMRU-6 Iquitos laboratory or transportation on dry ice to the NAMRU-6 laboratory facilities in Lima for serological testing (MNT, IgM) for DENV antibodies and cytokine testing. For samples collected with EDTA, a small aliquot of sample was removed for CBC and NS1 rapid diagnostic testing and the remaining sample was centrifuged and plasma was stored as described above for subsequent liver enzyme testing. Blood volumes depended on the study component.

### 2.1.2 Capillary Samples

If venipuncture was unacceptable to a study participant, we used aseptic techniques, retractable safety lancets and microtainers to collect blood from the tip of a finger. Our team was routinely able to collect as much as 2.0 ml (two 1.0 ml tubes without additive) for MNT testing of longitudinal samples and 1 ml (tubes with EDTA) from cluster contacts for NS1 rapid diagnostic testing and TaqMan PCR for DENV/ZIKV. Serum or plasma was separated and stored at -70°C in labeled Nunc polypropylene vials.

## 2.2 Other body fluids sampled for suspected Zika cases.

During the period of ZIKV transmission, we requested other types of biological samples from participants with suspected ZIKV infection. These samples were not collected systematically and IRB permission was not in place for the entire transmission period. When possible, however, other samples types listed below were requested from participants.

2.2.1 Urine Samples

A minimum urine volume of between 0.5-1.0 ml was collected by the subject in a sealed sterile screw capped vial, after cleaning of the meatus of the penis or labia of the vagina with a wipe and attempting a midstream collection.

### 2.2.2 Saliva sample collection

Universal transport medium with the soft applicator was used to sample the buccal mucosa. Applicator swabs were placed in the transport medium and broken off, leaving the swab portion in the medium.

### 2.2.3 Breast Milk and Semen.

A minimum of 0.5-1.0 ml expressed milk or semen was collected into sterilized urine collection cups. Before collections, we encouraged cleaning of the breasts, hands and penis ensuring there was no soap residue.

## 2.3 Sample Transport and storage

All specimen types were transported to our NAMRU-6 Iquitos laboratory in a cooler (2-8C). All acute disease cases and cluster contact samples were tested by either NS1 rapid diagnostic (DENV only) or real-time qRT-PCR within 24 hours of collection in our Iquitos facility. There were some exceptions but the principal goal of this project was to initiate mosquito feeding and blood sample collection on dengue and Zika virus infected individuals as soon as possible. Thus, sample arriving before 2pm were usually processed the same day and those after first thing the following morning. CBC testing was conducted at the time of sample processing. All remaining serum or plasma were stored at -80C for long term storage and subsequent transfer on dry ice to the NAMRU-6 laboratory in Lima. All ELISA and microneutralization testing was carried out in Lima.

# 3. Entomological surveys

Routine (~ 4-month intervals) *Aedes aegypti* surveys were carried out in the households participating in the surveillance cohort. Household mosquito abundance and productivity data were used (via the Data Core) for the development of mathematical and statistical models^10,11^. Adult *Ae. aegypti* collections from households during cluster investigations, when possible, were used to quantify exposure of viremic participants. Results from our previous studies in Iquitos^12–14^ indicate that the household is the appropriate spatial scale for estimating entomological risk for dengue, adult mosquito indices are the best predictors of dengue incidence, and immature indices (larvae and pupa) have the highest correlations with seroprevalence rates. We used the following well-established protocols to estimate population densities and calculate 22 *Ae. aegypti* density indices^2,12^.

## 3.1 Larval and pupal collections

We employed the pupal/demographic survey methodology that quantifies pupae and larvae infested containers.^15–19^ In each house, we inspected all water-holding containers following methods described previously.^12,13,20,21^ Our entomology team surveyed approximately 100 houses per day. To maintain quality control (QC), an entomological supervisor accompanied the survey team and a second supervisor reviewed data daily. Additionally, algorithms were developed within the database to identify potential reporting or data entry errors. Pupae collected from ten different locations during routine entomological surveys were used to establish distinct laboratory colonies to generate a genetically diverse laboratory strain (GDLS) for use in Project 1 for up to five mosquito feeding experiments per week.

3.2 Adult captures

Adult *Ae.aegypti* were collected with “Prokopack” aspirators,^22^ which collect up to 4.2 times more adults overall and six times more blood fed females than a CDC backpack aspirator.^23^ Labeled collections were transported to the field laboratory for identification and counts.

## 3.3 Establishment of the Genetically Diverse Laboratory *Aedes aegypti* strain

Mosquitoes used to assess human infectiousness were derived from local, wild type *Ae. aegypti*. A genetically diverse laboratory strain (GDLS)^24,25^ was established between August-December 2015 by collecting ~1,000 immature *Aedes aegypti* mosquitoes (males and females) from 10 locations representing a geographically stratified sample of the local, wild-type *Aedes aegypti* population across Iquitos. F_1_ eggs were used to generate the GDLS colony used for experimental feedings. All female F_0_ adults were screened for DENV or ZIKV by RT-PCR before hatching F_1_ eggs for that strain. No F_0_ adult mosquitoes tested positive for DENV or ZIKV RNA. The 10 geographically distinct Iquitos populations were considered DENV and ZIKV free. Each GDLS generation was used for approximately 6 months so that at the end of the study in April 2019 we were on the sixth generation of the GDLS.

The GDLS strategy avoids two potential problems: (1) it minimizes uncontrolled differences between experiments that may result by continuously using wild mosquitoes sampled from the field, whose genetic composition could shift temporally and (2) it maintains a level of genetic diversity that is representative of natural populations, which could be lost during laboratory colonization. The goal of the GDLS strategy was to recreate consistent representative wild-type genetic diversity across the 5 years of our vector competence experiments.


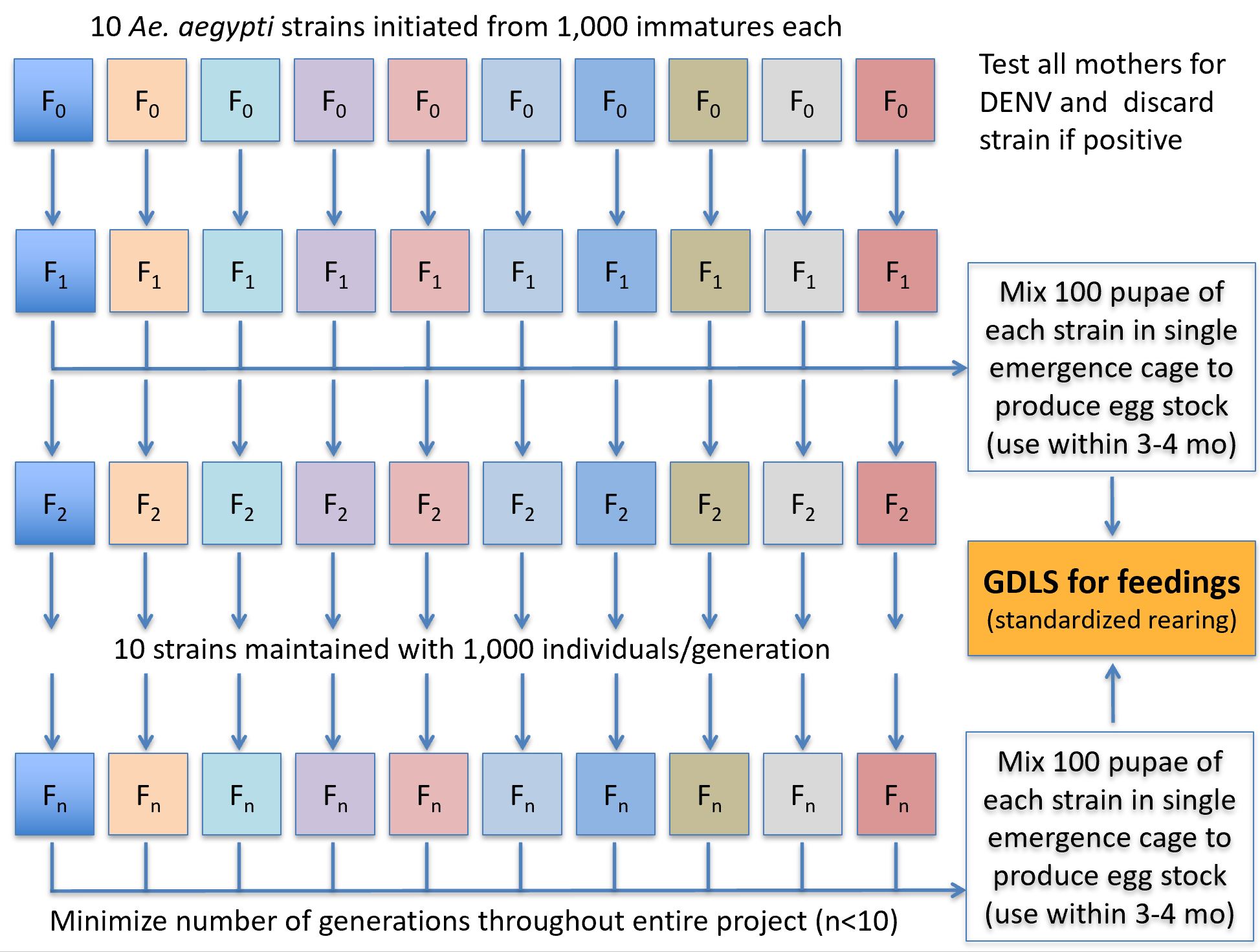


Figure S1

# 4. Laboratory Assays

TaqMan real-time PCR assays of human blood and mosquitoes were conducted in the NAMRU-6 Iquitos laboratory. In general, results from morning and afternoon runs were available in the early afternoon on the same day or early the next morning, respectively. As soon as PCR results were available, participants with positive results who had indicated willingness to participate in additional studies were immediately contacted and enrolled in the viremic participant protocol, and mosquito feeding and blood sample collection procedures were conducted. Participants who started the viremic participant procedures before having a PCR diagnosis were informed of their infection status within 24 hours and invited to repeat the procedures if they were confirmed as DENV or ZIKV positive. A complete blood count was obtained at NAMRU-6 Iquitos. All laboratory results were provided to study participants with an explanation by study personnel.

## 4.1 Taqman Real-Time PCR for DENV serotypes 1-4.

Viral RNA was extracted from whole blood and/or serum samples using QIAamp Viral RNA Mini Kits following the manufacturer’s guidelines. Using a method modified from Johnson et al.,^26^ primer and probe sets validated on an ABI 7500 real-time PCR platform (Applied Biosystems) were used with TaqMan Fast Virus 1-step RT-PCR master mix (Life Technologies) to detect DENV RNA in serum samples. The protocol consists of two assays: the first is a real-time multiplex assay that detects DENV-1, DENV-3, and DENV-4 and the second is a real-time singleplex assay that detects DENV-2.

## 4.2 Nested reverse transcription polymerase chain reaction (RT-PCR) for dengue serotypes 1–4.

Human samples with borderline cycle threshold values were confirmed positive or negative for DENV or ZIKV using previously extracted RNA and a nested RT-PCR protocol described by Lanciotti et al.^27^

## 4.3 Quantitative Real-Time PCR for ZIKV.

Viral RNA was extracted from serum samples using QIAamp Viral RNA Mini Kit (QIAGEN) according to the manufacturer's instructions. Assays were performed on the ABI 7500 real-time PCR platform (Applied Biosystems) using Superscript III Platinum One-Step qRT-PCR kits (ThermoFisher) in duplicate 25 ul final volume reactions comprised of 5 ul template, 12.5 ul of 2x reaction mix, 0.5 ul of 50 mM magnesium sulfate, 0.5 ul of SuperScript III RT/Platinum Taq mix, and 0.5 ul of 10 mM each primers and probe.^28^ Reactions were cycled under the following amplification conditions: 50°C for 15 min followed by 95°C for 2 min and 40 cycles of 95°C for 15 sec and 58°C for 30 sec.

## 4.4 IgM capture ELISA

Serum was tested for anti-DENV IgM antibody using a IgM capture ELISA NAMRU-6 protocol.^9,29^ Antibody in sera that tested positive was titered, and a 4-fold rise (from acute to convalescent) in titer was considered evidence of seroconversion and recent DENV infection. During the period from May 2016-April 2017 a rise in DENV IgM antibody was presumed to be seroconversion to ZIKV due to a Zika outbreak and undetectable DENV transmission throughout Iquitos^30^.

4.5 Serotype-specific microneutralization enzyme immunoassay (MNT).

We used a validated NAMRU-6 protocol adapted from Vorndam and Beltran,^31^ in which 96-well plates (TC-treated) were inoculated with Vero cells at 2 x 10^5^ cells/mL and then incubated at 37ºC, with 5% CO_2_ for two days or until the cell monolayer was confluent. Serum samples were inactivated at 56°C for 30 min, then serially diluted in triplicate in a two-fold series from 1:20 to 1:1280 on a 96-well plate, along with negative serum and positive hyperimmune mouse ascitic fluid (HMAF) controls. Diluted virus (dilution factor determined by NAMRU-6 validation assays) was mixed with inactivated sera and incubated at 4ºC overnight. A Vero cell suspension at 2x10⁵cells/mL in 10% Fetal Bovine Serum (FBS) Eagle’s Minimum Essential Medium (EMEM) was then added to each well with the serum-virus mixture and incubated at 37ºC with 5% CO_2_ for five days. After the five days, the cell culture supernatant was discarded and the cells were fixed with ethanol/methanol, washed with phosphate-buffered saline (PBS), blocked with skim milk, then anti-DENV HMAF was added and incubated for 2 h at 37ºC, washed with PBS, followed by addition of labeled goat anti-mouse IgG +IgM peroxidase and incubation for 1 h at 37ºC, washed with PBS again, and incubated at room temperature for one hour with 2,2′-azinobis(3-ethylbenzthiazoline-6-sulfonic acid) (ABTS) substrate.

Plates were read using an enzyme-linked immunosorbent assay (ELISA) reader (Microplate Reader Biotek Instruments Inc.) at 405 nm with a 630 nm reference filter. A cut-off value was established for each plate as the numeric value of 50% of the mean optical density (OD) of virus controls and the endpoint titer was the highest serum dilution with mean OD below the cut off value. Endpoint titers were reported as <1/40, 1/80, 1/160, …, ­>1/2560.

# 5. Direct Blood Feeding Experiments on DENV/ZIKV-positive participants

## 5.1 Blood feeding Procedures.

Mosquito feeding procedures were developed through a pilot study^32^ and acceptability was explored via focus groups.^33^ For each feeding, two containers of 30 (15 for children < 14 years of age) uninfected mosquitoes from a GDLS **(see SI section 3.3)** were placed on the arms or legs of a participant for a maximum of 10 minutes in their homes. The option to carry out the feeds in the NAMRU-6 laboratory was offered as an alternative. Containers were then transported in a secure container to the NAMRU-6 laboratory for processing.

## 5.2 Mosquito processing workflow

After participants had fed mosquitoes directly, the containers were placed in a −20°C freezer for 5 minutes to sedate mosquitoes before separating engorged from unfed mosquitoes. Two to three of the engorged mosquitoes were immediately frozen for testing to determine capillary blood meal virus titer by Fluorescent Focus Assay (FFA) and quantitative RT-PCR. The remaining engorged mosquitoes were kept in one-pint cardboard containers with mesh tops, provided a 10% sucrose solution, and held at 27°C for 7 or 14 days. Early experiments tested the mosquitoes at 14 days post infection (DPI), whereas later experiments in particular for ZIKV participants testing was done in two groups between day 7 and 14 DPI. Daily mortality was recorded and on the day of harvest up to 20 mosquitoes were forced to salivate into in a 10 µL filter tip containing 6 µL of a 1:1 of 30% sucrose and Heat Inactivated Fetal calf serum (HIFCS)^34^. Following *in vitro* salivation, heads and bodies from each mosquito that had fed on a study participant were stored at −80°C for future testing. The saliva solution was immediately inoculated intrathoracically into 5 DENV/ZIKV-naïve mosquitoes.^35^ The mosquitoes that shared a saliva inoculum from the same mosquito were pooled. After 7 days of incubation, inoculated mosquitoes were harvested and RNA was extracted from the inoculated mosquito pools. Each pool assayed saliva from a single mosquito that had fed on a viremic participant. Aliquots from each of the ~20 inoculated mosquito pools, each representing the 20 mosquitoes that fed on a given viremic participants, were combined and tested for DENV or ZIKV by quantitative RT-PCR. If the pool of RNA extract was negative, the participant was considered not infectious to mosquitoes and no further mosquito testing was performed. If the pool was positive, RNA samples from each of the 20 mosquitoes was individually tested by quantitative RT-PCR to determine the percentage of mosquitoes with infectious saliva.

# 6. Project 2 Interviews and Procedures

## 6.1 Full Retrospective Movement Survey

The full retrospective movement survey (full RMS) was developed through a formative research process, validated by comparing results from the RMS to GPS data collected in a previous study,^36^ and described fully in other publications.^36^ This semi-structured interview asked respondents to recall to their past two weeks and report locations visited in that time period. Locations asked about included communal sites such as local markets or schools or health facilities. Interviewers focused on obtaining more detail about residential or multi-use type locations, including time spent there (frequency and duration), type of activity while there (i.e., sitting vs. moving around), as well as included a detailed description on how to find the house in addition to any address information available. The full RMS was applied to index cases on the day of enrollment and, if index was positive for DENV or ZIKV, triggered contact cluster investigations.

6.2 24-hour RMS (“24hr RMS”)

Adapted from the full RMS, this survey was applied daily to DENV- or ZIKV-positive index or cluster participants, to assess daily changes in the amount and type of movement.^37^

6.3 Dengue Illness Perceptions and Responses (IPR) survey

Developed through a formative research process (focus groups, medical expert review, literature review, and pilot process), the IPR survey probed participants on the full range of symptoms experienced, and then asked for characterization of most of the symptoms; i.e., intensity, duration, frequency, description, and what medications/actions mitigated symptoms.^38^

6.4 Health Related Quality of Life (HRQoL) survey

To assess the impact of dengue or Zika illness on overall quality of life^39^ we used the validated HRQoL survey, the Quality of Well-Being – Self-Administered (QWB-SA) scale, which was developed for use with chronic conditions (<https://hoap.ucsd.edu/qwb-info/QWB-Manual.pdf>).

6.5 Costs survey

To assess the types of expenditures that dengue patients and their families incurred during illness, we used a short survey (i.e., medications, transportation to facilities, days lost from work) on the last day of illness (once patient was PCR negative) and 30 days after the first symptom. The survey was previously used in a different region of Peru in an endemic dengue setting.^40^

## 6.6 GPS monitoring

We used a GPS data-logger ("Igot-U GT100", Mobile Action Technology Inc., Taiwan) at all times for a 10 d period (to capture movement during both viremic and “healthy” periods)^6,36^ Prior to enrollment, a field technician explained the purpose of the study and handed the participant a pamphlet addressing potential concerns about GPS technology^5^. Participants were provided chargers or staff provided freshly charged units every 3-4 days.

## 6.7 Temperature Monitoring

Temperature, vital signs, and tourniquet test was performed daily while study participants were viremic.

# 7. References

1. Stoddard ST, Forshey BM, Morrison AC, et al. House-to-house human movement drives dengue virus transmission. Proc Natl Acad Sci U S A 2013;110(3):994–9.

2. Cromwell EA, Stoddard ST, Barker CM, et al. The relationship between entomological indicators of Aedes aegypti abundance and dengue virus infection. PLoS Negl Trop Dis 2017;11(3):e0005429.

3. Forshey BM, Reiner RC, Olkowski S, et al. Incomplete Protection against Dengue Virus Type 2 Re-infection in Peru. PLoS Negl Trop Dis 2016;10(2):e0004398.

4. Olkowski S, Forshey BM, Stoddard S, et al. Effects of pre-existing DENV antibody on the occurrence of symptomatic illness associated with novel DENV-4 infection. In: American Society of Tropical Medicine and Hygeine. 2011.

5. Paz-Soldan VA, Stoddard ST, Vazquez-Prokopec G, et al. Assessing and maximizing the acceptability of global positioning system device use for studying the role of human movement in dengue virus transmission in Iquitos, Peru. Am J Trop Med Hyg 2010;82(4):723–30.

6. Vazquez-Prokopec GM, Bisanzio D, Stoddard ST, et al. Using GPS technology to quantify human mobility, dynamic contacts and infectious disease dynamics in a resource-poor urban environment. PLoS One 2013;8(4):e58802.

7. Paz-Soldan VA, Yukich J, Soonthorndhada A, et al. Design and Testing of Novel Lethal Ovitrap to Reduce Populations of Aedes Mosquitoes: Community-Based Participatory Research between Industry, Academia and Communities in Peru and Thailand. PLoS One 2016;11(8):e0160386.

8. Morrison AC, Reiner RC Jr, Elson WH, et al. Efficacy of a spatial repellent for control of Aedes-borne virus transmission: A cluster randomized trial in Iquitos, Peru [Internet]. bioRxiv. 2021;Available from: http://dx.doi.org/10.1101/2021.03.03.21252148

9. Forshey BM, Guevara C, Laguna-Torres VA, et al. Arboviral etiologies of acute febrile illnesses in Western South America, 2000-2007. PLoS Negl Trop Dis 2010;4(8):e787.

10. Reiner RC Jr, Stoddard ST, Vazquez-Prokopec GM, et al. Estimating the impact of city-wide Aedes aegypti population control: An observational study in Iquitos, Peru. PLoS Negl Trop Dis 2019;13(5):e0007255.

11. Cavany SM, España G, Lloyd AL, et al. Optimizing the deployment of ultra-low volume and targeted indoor residual spraying for dengue outbreak response. PLoS Comput Biol 2020;16(4):e1007743.

12. Morrison AC, Gray K, Getis A, et al. Temporal and geographic patterns of Aedes aegypti (Diptera: Culicidae) production in Iquitos, Peru. J Med Entomol 2004;41(6):1123–42.

13. Getis A, Morrison AC, Gray K, Scott TW. Characteristics of the spatial pattern of the dengue vector, Aedes aegypti, in Iquitos, Peru. Am J Trop Med Hyg 2003;69(5):494–505.

14. Gunning CE, Okamoto K, Astete H, et al. Efficacy of Aedes aegypti control by indoor Ultra Low Volume (ULV) insecticide spraying in Iquitos, Peru. PLoS Negl Trop Dis 2018;12(4):e0006378.

15. Focks DA, Haile DG, Daniels E, Mount GA. Dynamic life table model for Aedes aegypti (Diptera: Culicidae): analysis of the literature and model development. J Med Entomol 1993;30(6):1003–17.

16. Focks DA, Haile DG, Daniels E, Mount GA. Dynamic life table model for Aedes aegypti (diptera: Culicidae): simulation results and validation. J Med Entomol 1993;30(6):1018–28.

17. Focks DA, Chadee DD. Pupal survey: an epidemiologically significant surveillance method for Aedes aegypti: an example using data from Trinidad. Am J Trop Med Hyg 1997;56(2):159–67.

18. Focks DA, Sackett SR, Bailey DL, Dame DA. Observations on container-breeding mosquitoes in New Orleans, Louisiana, with an estimate of the population density of Aedes aegypti (L.). Am J Trop Med Hyg 1981;30(6):1329–35.

19. Southwood TR, Murdie G, Yasuno M, Tonn RJ, Reader PM. Studies on the life budget of Aedes aegypti in Wat Samphaya, Bangkok, Thailand. Bull World Health Organ 1972;46(2):211–26.

20. Morrison AC, Astete H, Chapilliquen F, et al. Evaluation of a sampling methodology for rapid assessment of Aedes aegypti infestation levels in Iquitos, Peru. J Med Entomol 2004;41(3):502–10.

21. Schneider JR, Morrison AC, Astete H, Scott TW, Wilson ML. Adult size and distribution of Aedes aegypti (Diptera: Culicidae) associated with larval habitats in Iquitos, Peru. J Med Entomol 2004;41(4):634–42.

22. Vazquez-Prokopec GM, Galvin WA, Kelly R, Kitron U. A new, cost-effective, battery-powered aspirator for adult mosquito collections. J Med Entomol 2009;46(6):1256–9.

23. Clark GG, Seda H, Gubler DJ. Use of the “CDC backpack aspirator” for surveillance of Aedes aegypti in San Juan, Puerto Rico. J Am Mosq Control Assoc 1994;10(1):119–24.

24. Wise de Valdez MR, Suchman EL, Carlson JO, Black WC. A large scale laboratory cage trial of Aedes densonucleosis virus (AeDNV). J Med Entomol 2010;47(3):392–9.

25. Wise de Valdez MR, Nimmo D, Betz J, et al. Genetic elimination of dengue vector mosquitoes. Proc Natl Acad Sci U S A 2011;108(12):4772–5.

26. Johnson BW, Russell BJ, Lanciotti RS. Serotype-specific detection of dengue viruses in a fourplex real-time reverse transcriptase PCR assay. J Clin Microbiol 2005;43(10):4977–83.

27. Lanciotti RS, Calisher CH, Gubler DJ, Chang GJ, Vorndam AV. Rapid detection and typing of dengue viruses from clinical samples by using reverse transcriptase-polymerase chain reaction. J Clin Microbiol 1992;30(3):545–51.

28. Garcia Glaessner A, Barrera P, Morrison AC, Scott TW, Leguia M. ZIKA VIRUS DETECTION IN PREVIOUSLY UNDIAGNOSABLE SAMPLES: OPTIMIZATION OF A QUANTITATIVE RT-PCR ASSAY FOR SAMPLES OF LOW VIRAL CONCENTRATION. In: AMERICAN JOURNAL OF TROPICAL MEDICINE AND HYGIENE. AMER SOC TROP MED & HYGIENE 8000 WESTPARK DR, STE 130, MCLEAN, VA 22101 USA; 2019. p. 63–63.

29. Morrison AC, Minnick SL, Rocha C, et al. Epidemiology of dengue virus in Iquitos, Peru 1999 to 2005: interepidemic and epidemic patterns of transmission. PLoS Negl Trop Dis 2010;4(5):e670.

30. Weldon CT, Riley-Powell AR, Aguerre IM, et al. “Zika is everywhere”: A qualitative exploration of knowledge, attitudes and practices towards Zika virus among women of reproductive age in Iquitos, Peru. PLoS Negl Trop Dis 2018;12(8):e0006708.

31. Vorndam V, Beltran M. Enzyme-linked immunosorbent assay-format microneutralization test for dengue viruses. Am J Trop Med Hyg 2002;66(2):208–12.

32. Long KC, Sulca J, Bazan I, et al. Feasibility of feeding Aedes aegypti mosquitoes on dengue virus-infected human volunteers for vector competence studies in Iquitos, Peru. PLoS Negl Trop Dis 2019;13(2):e0007116.

33. Morrison AC, Schwarz J, Long KC, et al. Acceptability of Aedes aegypti blood feeding on dengue virus-infected human volunteers for vector competence studies in Iquitos, Peru. PLoS Negl Trop Dis 2019;13(2):e0007090.

34. Aitken THG. An in vitro feeding technique for artificially demonstrating virus transmission by mosquitoes. J Am Mosq Control Assoc 1977;37(1):130–3.

35. Nguyet MN, Duong TH, Trung VT, et al. Host and viral features of human dengue cases shape the population of infected and infectious Aedes aegypti mosquitoes. Proc Natl Acad Sci U S A 2013;110(22):9072–7.

36. Paz-Soldan VA, Reiner RC Jr, Morrison AC, et al. Strengths and weaknesses of Global Positioning System (GPS) data-loggers and semi-structured interviews for capturing fine-scale human mobility: findings from Iquitos, Peru. PLoS Negl Trop Dis 2014;8(6):e2888.

37. Schaber KL, Paz-Soldan VA, Morrison AC, et al. Dengue illness impacts daily human mobility patterns in Iquitos, Peru. PLoS Negl Trop Dis 2019;13(9):e0007756.

38. Elson WH, Reiner RC, Siles C, et al. Heterogeneity of dengue illness in community-based prospective study, Iquitos, Peru. Emerg Infect Dis 2020;26(9):2077–86.

39. Elson WH, Riley-Powell AR, Morrison AC, et al. Measuring health related quality of life for dengue patients in Iquitos, Peru. PLoS Negl Trop Dis 2020;14(7):e0008477.

40. Salmon-Mulanovich G, Blazes DL, Lescano AG, Bausch DG, Montgomery JM, Pan WK. Economic burden of dengue virus infection at the household level among residents of Puerto Maldonado, Peru. Am J Trop Med Hyg 2015;93(4):684–90.
